# Supplementary material for: Artificial Intelligent Deep Learning Molecular Generative Modeling of Scaffold-Focused and Cannabinoid CB2 Target-Specific Small-Molecule Sublibraries
Source: Cells. 2022 Mar 7;11(5):915. doi: 10.3390/cells11050915 (PMC8909864; doi:10.3390/cells11050915)

Supplementary Table S1. The architecture of the g-DeepMGM with 256 units for each LSTM layer

```
_________________________________________________________________
Layer (type)                 Output Shape              Param #
=================================================================
lstm_3 (LSTM)                (None, 30, 256)           292864
_________________________________________________________________
dropout_2 (Dropout)          (None, 30, 256)           0
_________________________________________________________________
lstm_4 (LSTM)                (None, 256)               525312
_________________________________________________________________
dense_2 (Dense)              (None, 29)                7453
=================================================================
Total params: 825,629
Trainable params: 825,629
Non-trainable params: 0
```

Supplementary Table S2. The architecture of the g-DeepMGM with 512 units for each LSTM layer

Layer (type)	Output Shape	Param #
lstm_3 (LSTM)	(None, 30, 512)	1110016
dropout_2 (Dropout)	(None, 30, 512)	0
lstm_4 (LSTM)	(None, 512)	2099200
dense_2 (Dense)	(None, 29)	14877

Total params: 3,224,093
Trainable params: 3,224,093
Non-trainable params: 0

Supplementary Table S3. The validity, uniqueness, and novelty of sampled purine molecules under different epochs and sampling temperatures of the g-DeepMGM

Epoch	Temperature	Validity	Uniqueness	Novelty
**10** (training loss: 0.6309)	0.5	0.873	0.197	0.291
	**1.0**	**0.499**	**0.441**	**0.601**
	1.2	0.391	0.514	0.557
	1.5	0.338	0.506	0.731
**20** (training loss: 0.5966)	0.5	0.895	0.228	0.275
	**1.0**	**0.560**	**0.695**	**0.530**
	1.2	0.447	0.676	0.599
	1.5	0.331	0.689	0.658
**40** (training loss: 0.5700)	0.5	0.892	0.217	0.211
	**1.0**	**0.681**	**0.498**	**0.442**
	1.2	0.556	0.597	0.518
	1.5	0.406	0.635	0.628
**100** (training loss: 0.5444)	0.5	0.785	0.158	0.282
	**1.0**	**0.594**	**0.475**	**0.426**
	1.2	0.475	0.564	0.474
	1.5	0.369	0.588	0.664

Supplementary Table S4. The metrics for the MLP Discriminator

Algorithms	AUC	F1_score	ACC	Cohen-Kappa	MCC	precision	recall
**MLP_3_166_0**	0.936	0.558	0.912	0.516	0.556	0.417	0.846
**MLP_3_166_1**	0.920	0.471	0.871	0.416	0.487	0.320	0.891
**MLP_3_166_2**	0.907	0.487	0.902	0.439	0.469	0.368	0.719
**MLP_3_166_3**	0.935	0.512	0.917	0.469	0.486	0.413	0.672
**MLP_3_166_4**	0.909	0.435	0.859	0.376	0.445	0.293	0.844
**MLP_3_166_5**	0.940	0.514	0.910	0.469	0.497	0.395	0.734

Supplementary Figure S1. Log-likelihood and Wasserstein distance at different training epochs.

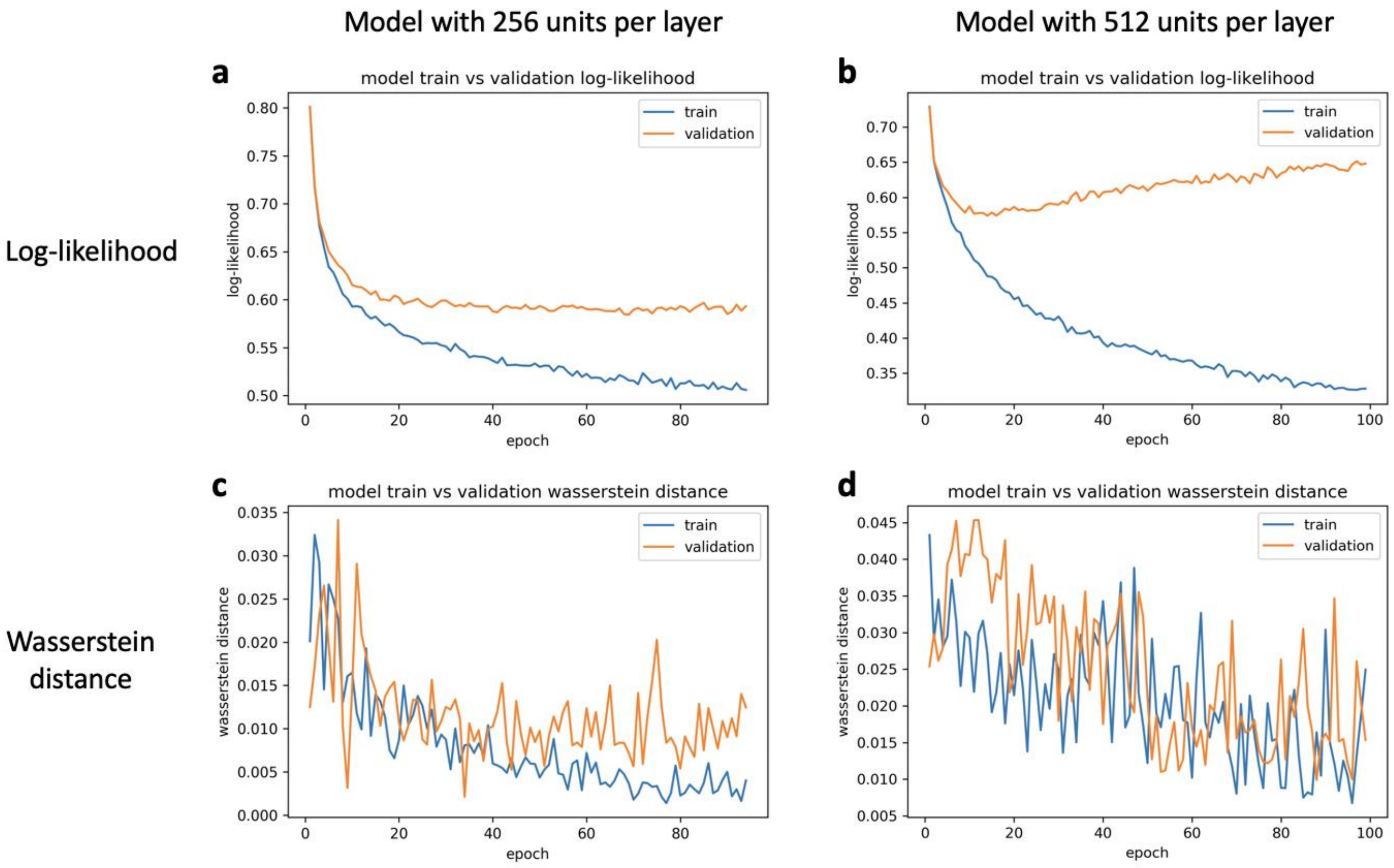

Log-likelihood during the training of the model with 256 units per layer (**a**) and 512 units per layer (**b**). Wasserstein distance during the training of the model with 256 units per layer (**c**) and 512 units per layer (**d**).

Supplementary Figure S2. Synthetic accessibility of generated indole molecules

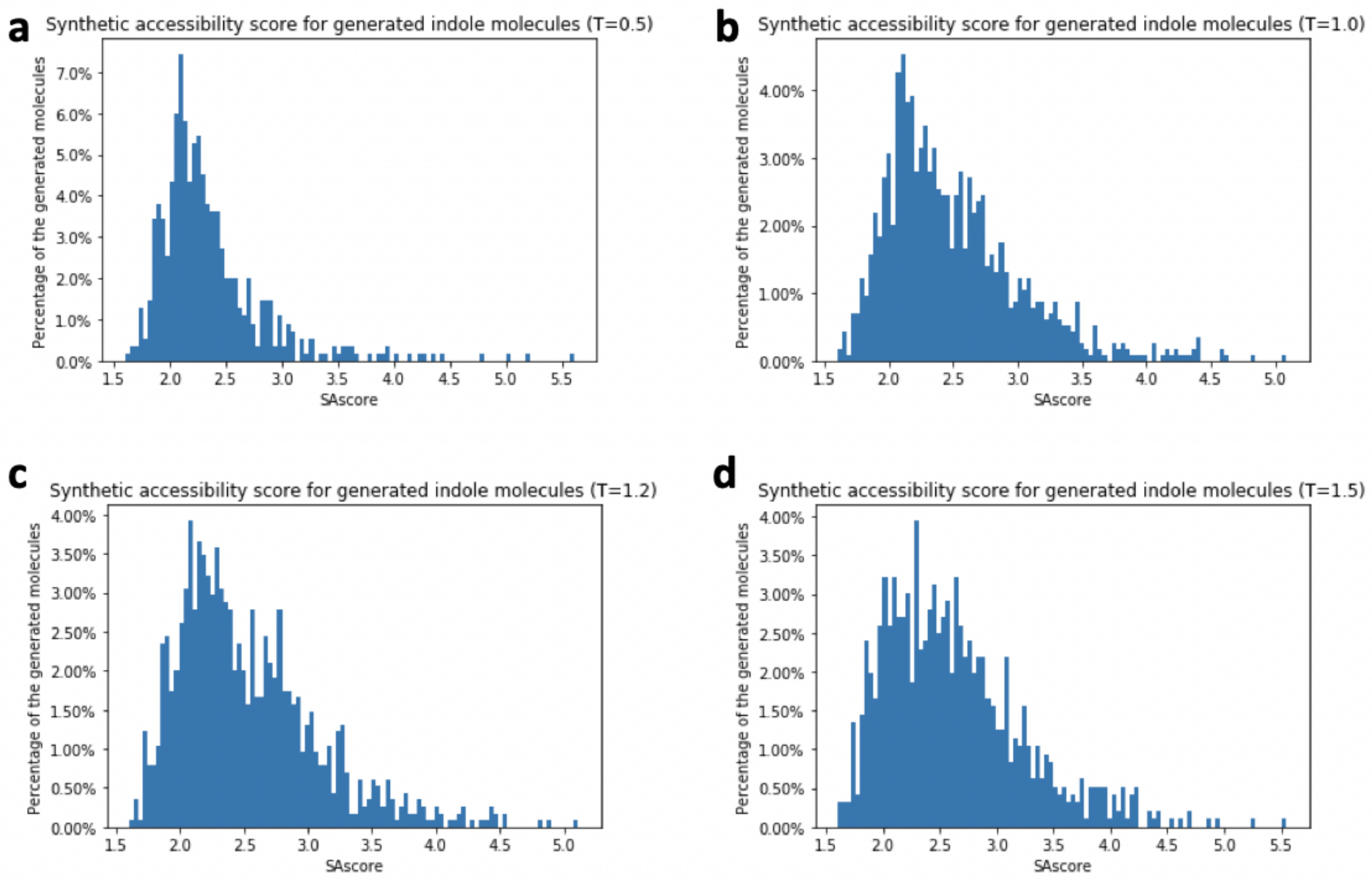

Synthetic accessibility scores for generated indole molecules when T=0.5 (**a**), T=1.0 (**b**), T=1.2 (**c**), and T=1.5 (**d**).

Supplementary Figure S3. Quantitative estimate of druglikeness of generated indole molecules

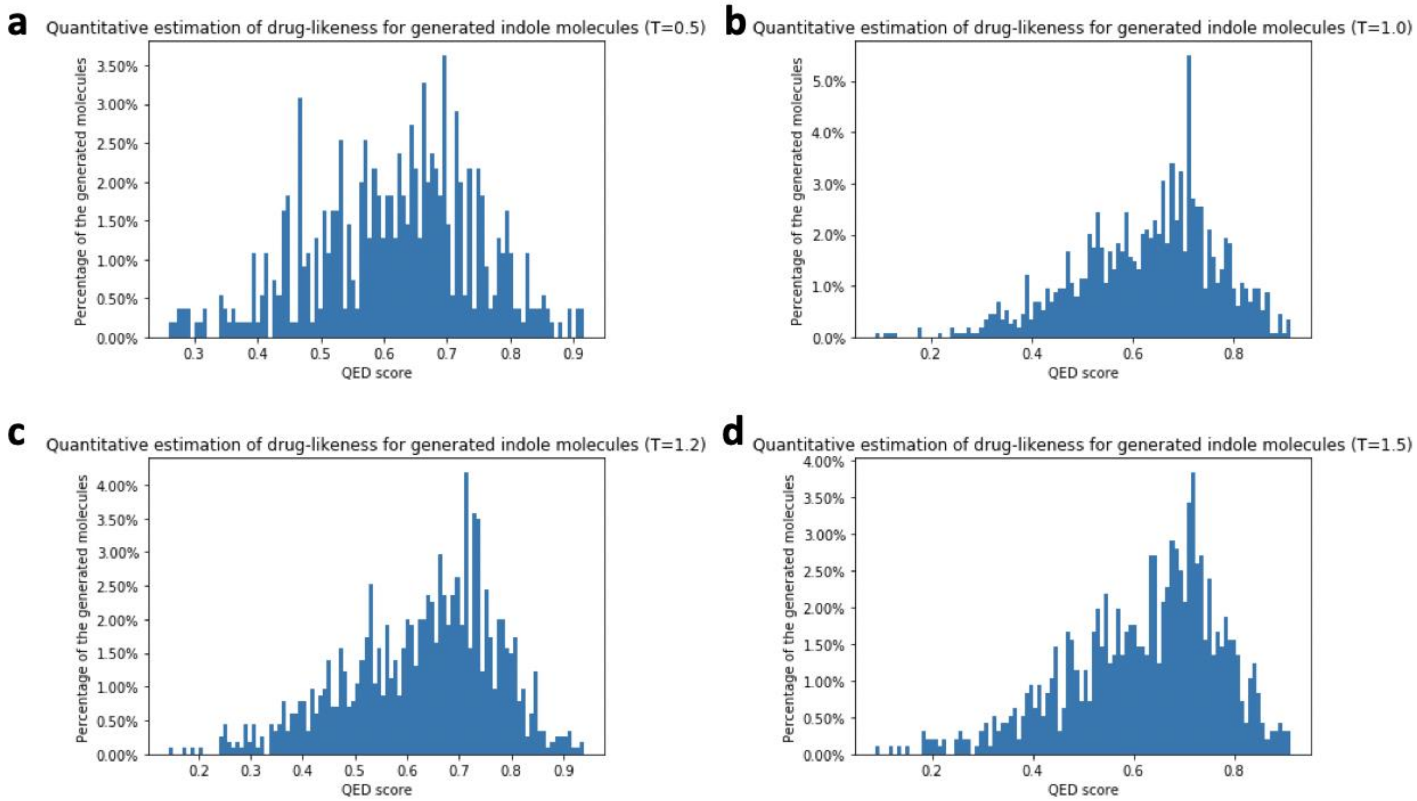

Quantitative estimation of drug-likeness for generated indole molecules when T=0.5 (**a**), T=1.0 (**b**), T=1.2 (**c**), and T=1.5 (**d**).

Supplementary Figure S4. Scree plots for physical-chemical properties and MACCS fingerprints

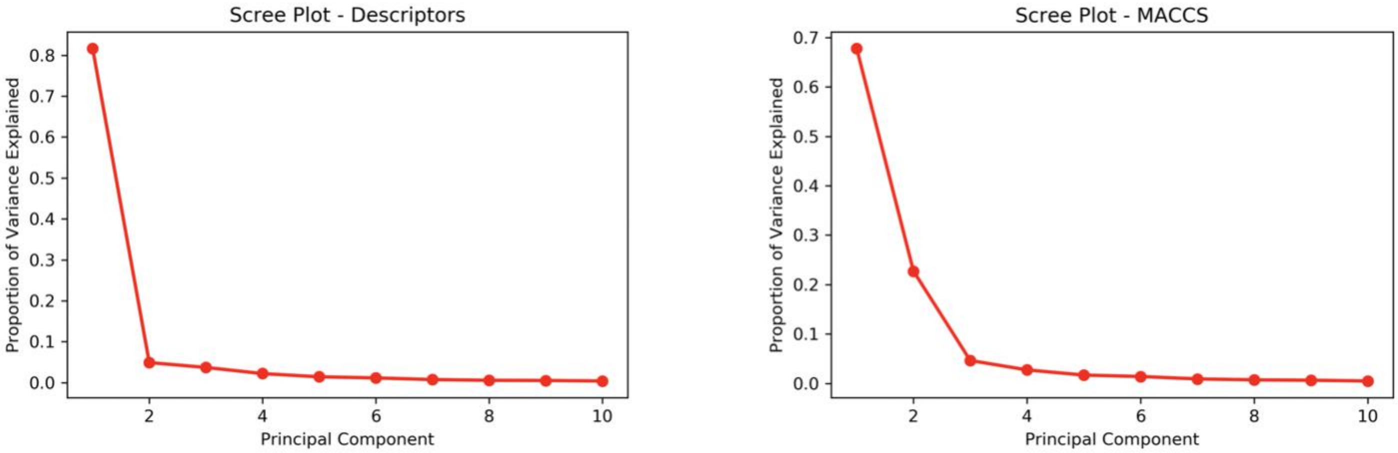

Supplementary Figure S5. Physical-chemical properties-based t-SNE analysis on generated indole molecules and training compounds.

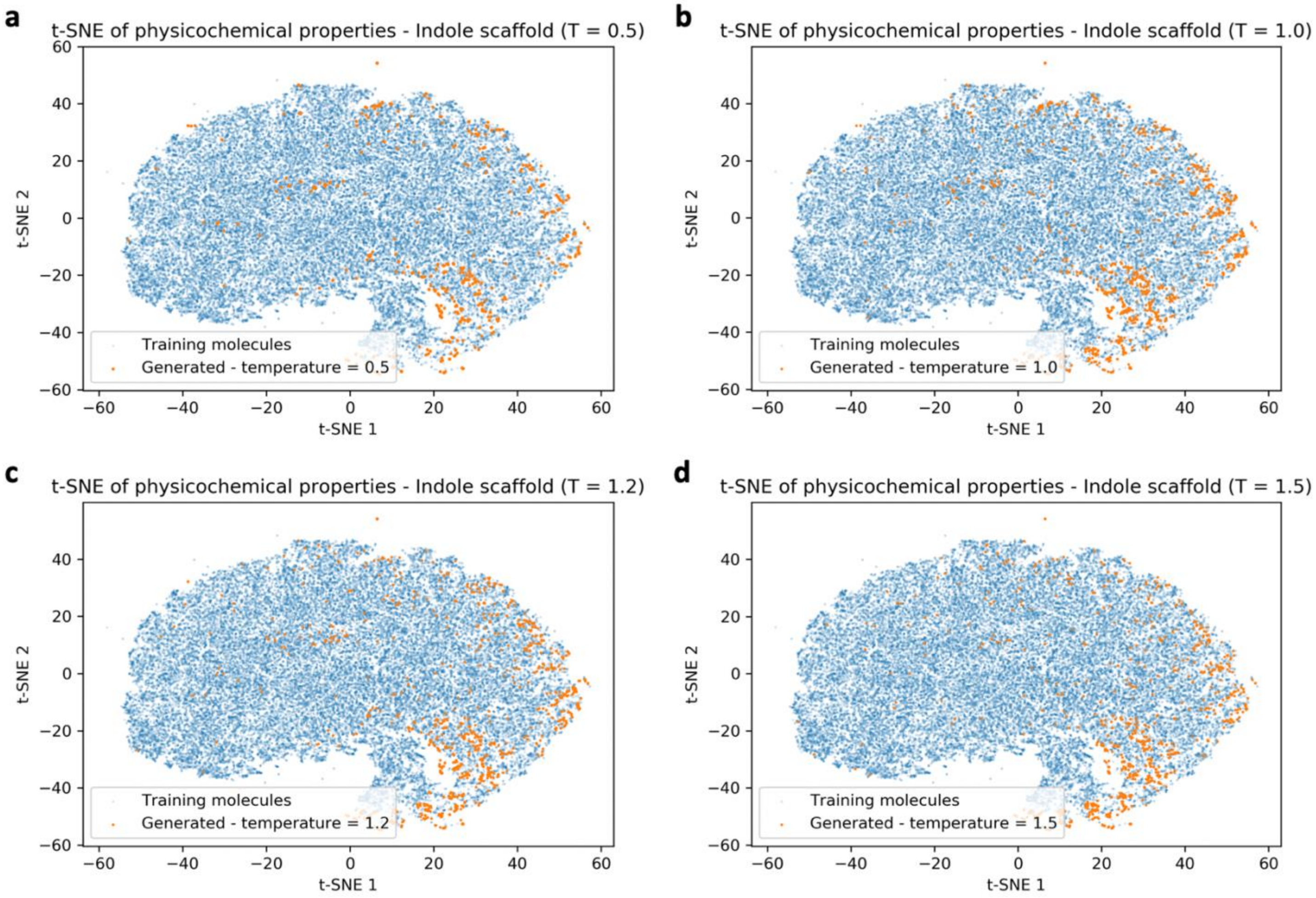

t-SNE of physicochemical properties for generated indole molecules when T=0.5 (**a**), T=1.0 (**b**), T=1.2 (**c**), and T=1.5 (**d**).

Supplementary Figure S6. MACCS fingerprint-based t-SNE analysis on generated indole molecules and training compounds.

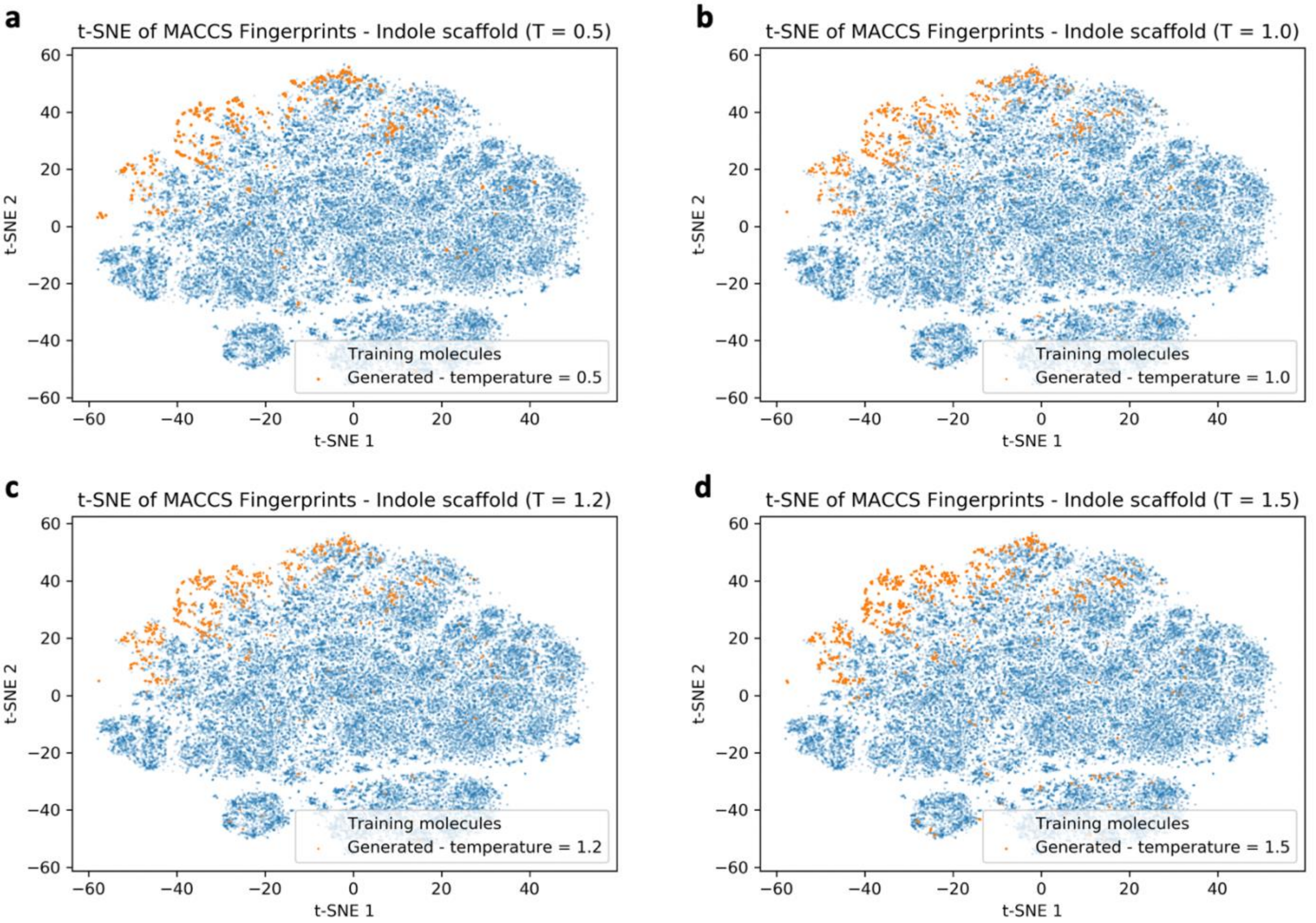

t-SNE of MACCS fingerprints for generated indole molecules when T=0.5 (**a**), T=1.0 (**b**), T=1.2 (**c**), and T=1.5 (**d**).

Supplementary application of g-DeepMGM on purine scaffold compounds generation

To further investigate the generation of the scaffold-focused chemical library with the g-DeepMGM, another privileged scaffold, purine, was selected for evaluation.

Similarly, the g-DeepMGM at four training epochs, 10, 20, 40, and 100 were selected for independent molecular sampling under four temperatures, 0.5, 1.0, 1.2, and 1.5. The purine scaffold has four possible positions for adding moieties. 2000 SMILES strings were sampled for each addition position under every epoch and sampling temperature. Totally 128,000 strings were sampled. The ability of the g-DeepMGM to generate valid SMILES strings peaked around the epoch 40 (**Supplementary Tab. S3**). A lower temperature usually results in high validity and low uniqueness and novelty, while a high temperature is inclined to sample unique and novel strings. The observation is consistent with the previous practice on the indole scaffold.

Supplementary Figure S7a demonstrates examples of generation outcomes of the g-DeepMGM at epoch 40 under each sampling temperature for four addition positions of the purine. Under the temperature 0.5, some special moieties including connecting a fluorobenzene group to piperazine can be seen. The observation of fluorine also suggests that atom characters besides “C,” “N,” and “O” can be predicted with the g-DeepMGM even under a relatively low sampling temperature. Under the temperature 1.0, interestingly, a nitrile is sampled with a cyano group connected to a cyclopentadiene group. A 5-methylhexyl group is spotted. It is common to spot sampled aliphatic carbon chains even under a higher sampling temperature. Under the temperature 1.2, the structure with positively charged nitrogen and negatively charged oxygen was sampled. Diversified sub-structural moieties including isoxazolidine, amide group, pyridine, and benzimidazole are perceived. Finally, under the temperature 1.5, linear structures such as carbon-carbon triple bonds were sampled. The inclusion of bromine and furan further contributes to the structural diversity with increased uniqueness and novelty. A column of reported similar compounds to the generated molecules under the sampling temperature 1.5 are listed for structural comparison.

Generated purine molecules from four addition positions at epoch 40 were combined. Again, the t-SNE analysis was performed to compare generated purine molecules and a half-million training compounds from the perspective of physical-chemical properties (**Supplementary Fig. S7b**) and MACCS fingerprints

(**Supplementary Fig. S7c**). Blue dots represent features of training compounds after the t-SNE dimension reduction, while colorful dots come from generated purine molecules. Colorful dots are distributed within the space covered by blue dots on both t-SNE plots. Certain chemical space is favored by generated purine molecules while most of the remaining space is uninviting. T-SNE plots for compounds generated at each temperature are supplied in **Supplementary Figure S8 and S9.** The g-DeepMGM was trained to compose SMILES of drug-like molecules starting with the input strings. Using the purine scaffold as the input to build a purine-focused library results in a chemical collection that is different from general drug-like compounds with an emphasis on specific physical-chemical and structural properties.

Supplementary Figure S7. Sampling examples on purine scaffold and t-SNE analysis of training set and generate purine molecules.

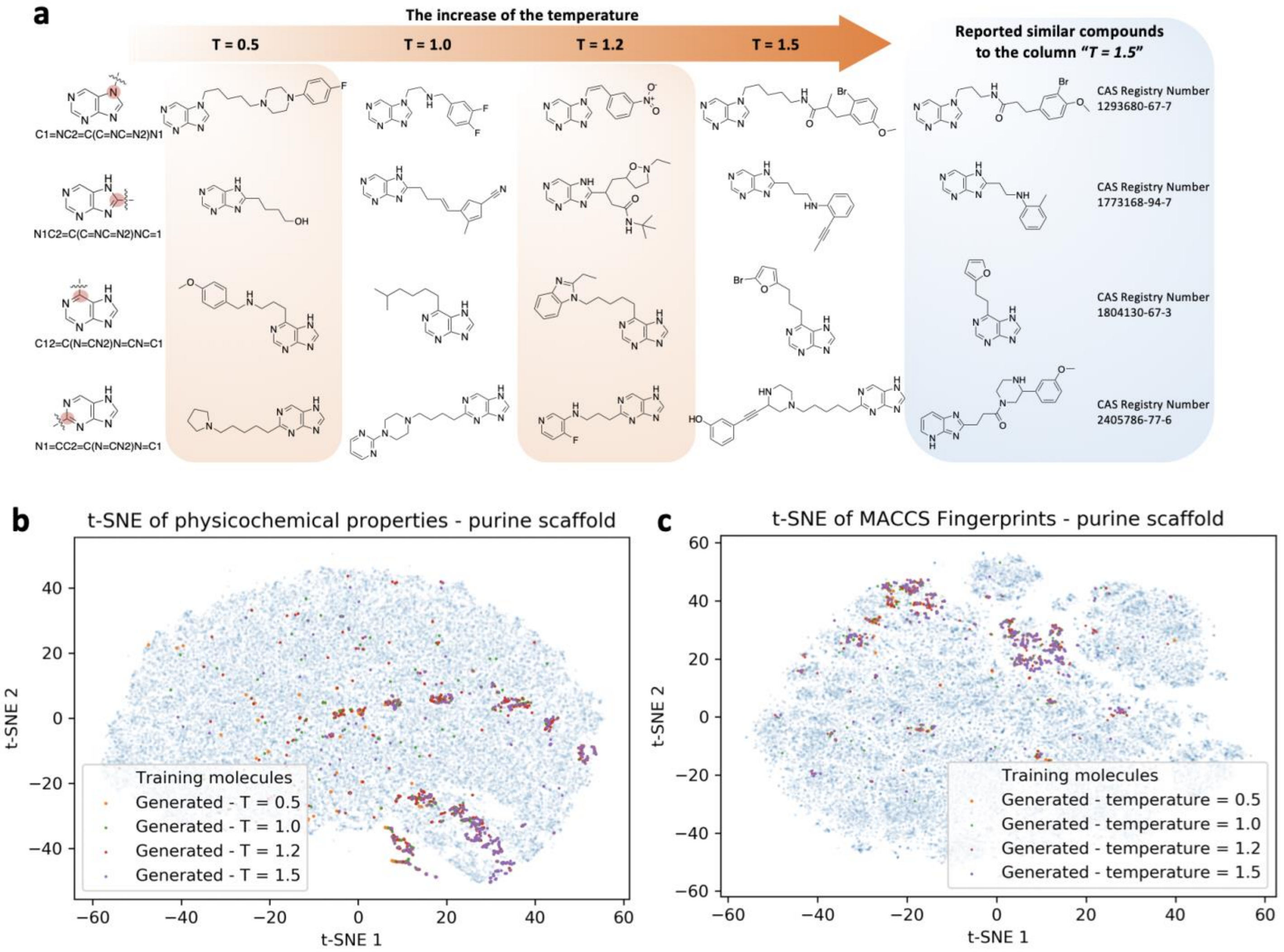

a Randomly selected sampling outcome for the purine scaffold under four temperatures with the g-DeepMGM at epoch 40. Four SMILES strings representing four addition positions on the purine were fed as the initial input. Reported similar compounds to the generated molecules in the column "T=1.5" are listed for comparison. Using both physical-chemical properties-based (**b**) and MACCS fingerprints-based (**c**) t-SNE analysis to compare generated purine molecules and training compounds.

Supplementary Figure S8. Physical-chemical properties-based t-SNE analysis on generated purine molecules and training compounds.

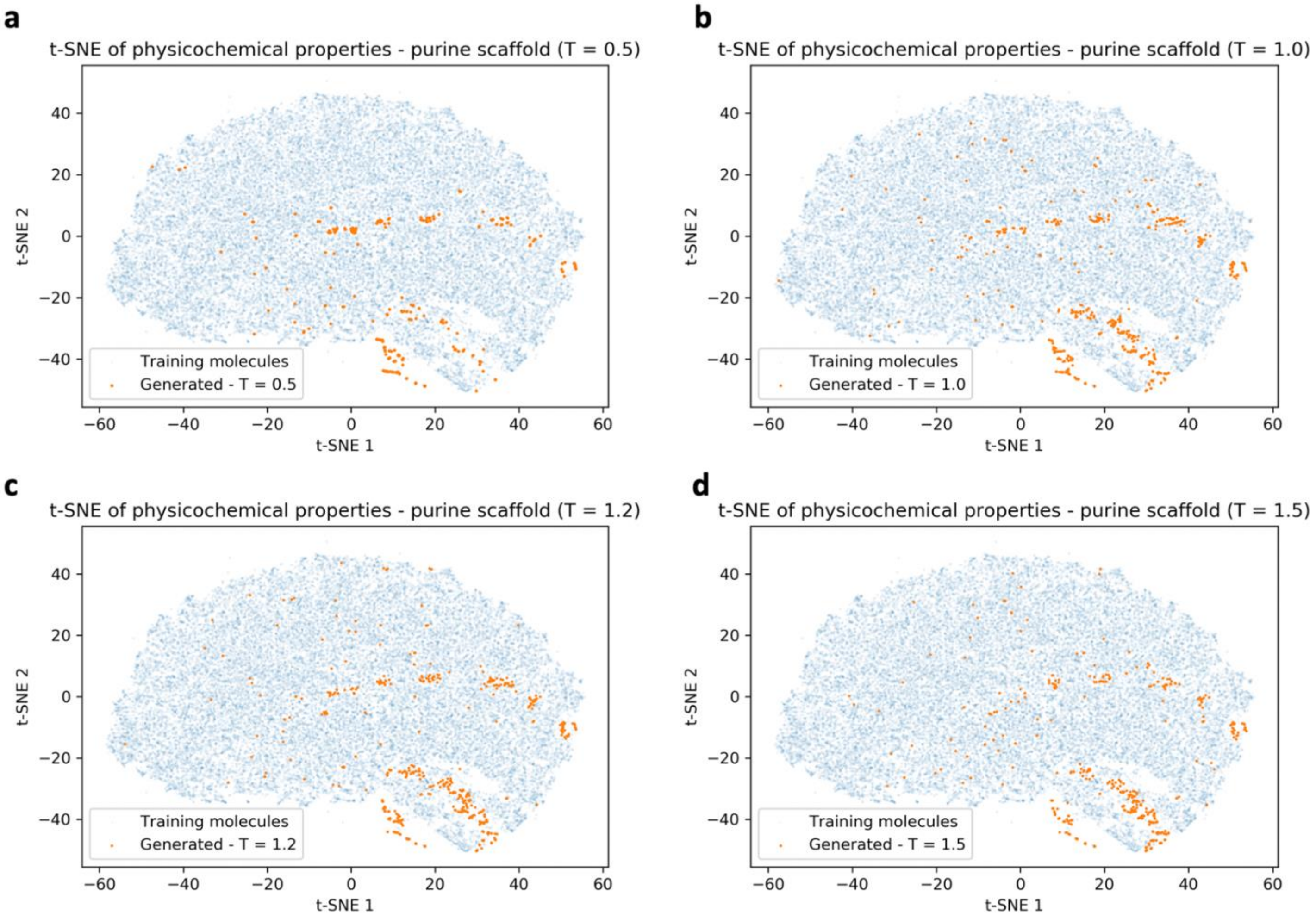

t-SNE of physicochemical properties for generated purine molecules when T=0.5 (**a**), T=1.0 (**b**), T=1.2 (**c**), and T=1.5 (**d**).

Supplementary Figure S9. MACCS fingerprint-based t-SNE analysis on generated purine molecules and training compounds.

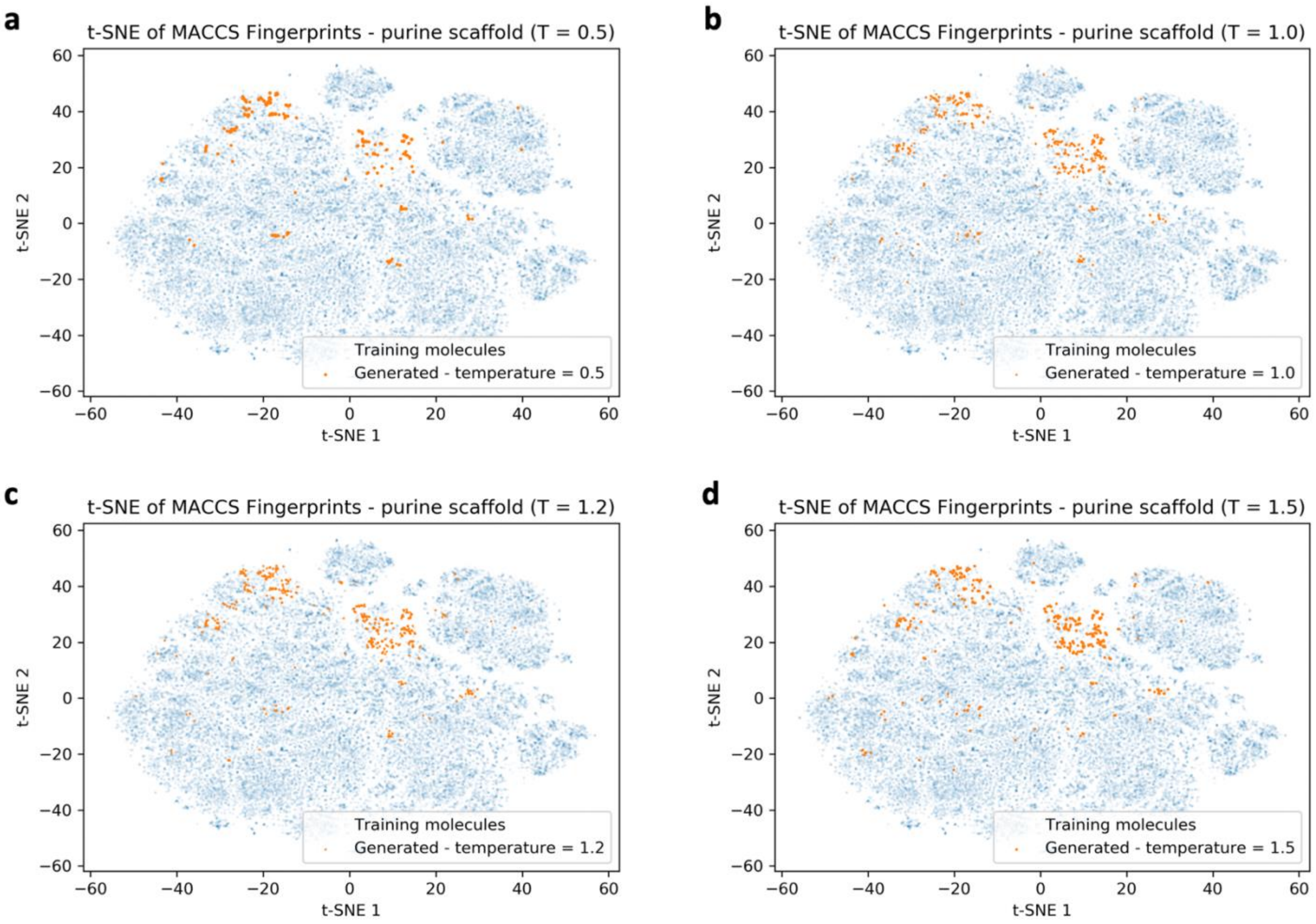

t-SNE of MACCS fingerprints for generated purine molecules when T=0.5 (**a**), T=1.0 (**b**), T=1.2 (**c**), and T=1.5 (**d**).

Supplementary Figure S10. Transfer learning strategies

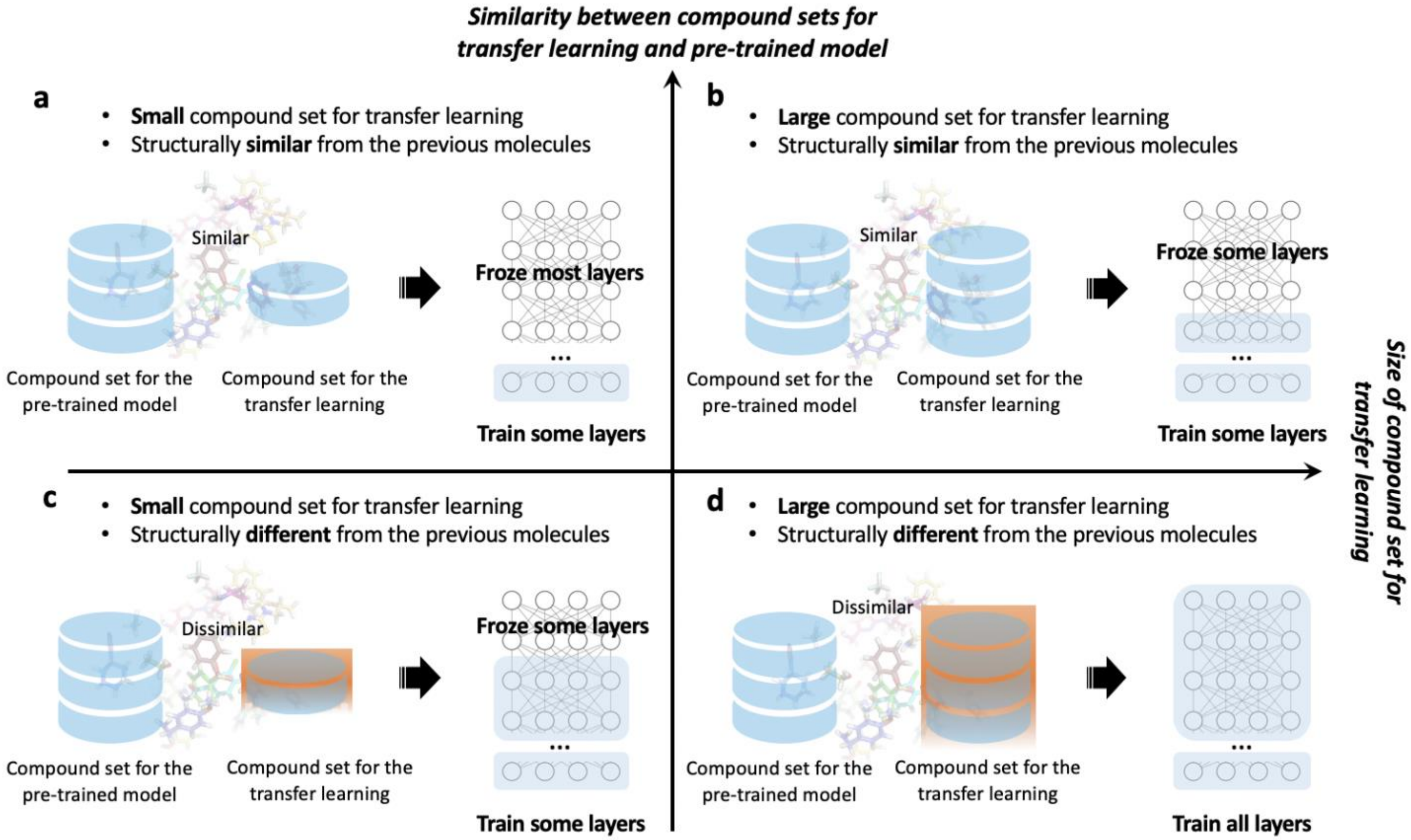

Transfer learning strategies under four scenarios: **a.** Small compound set for transfer learning with similar data structure to previous molecules. **b**. Large compound set for transfer learning with similar data structure to previous molecules. **c**. Small compound set for transfer learning with different data structure to previous molecules. **d.** Large compound set for transfer learning with different data structure to previous molecules.

Supplementary Figure S11. SAscore and QED for generated CB2 molecules

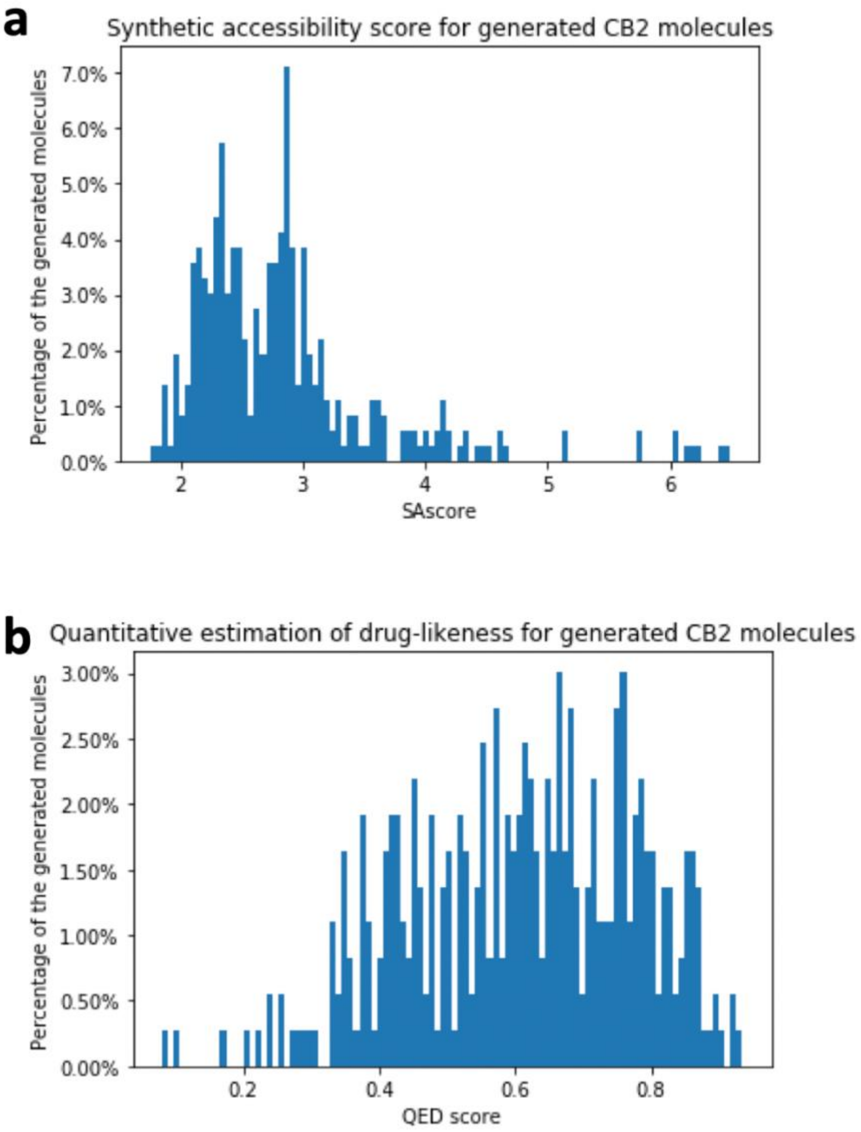

Synthetic accessibility (**a**) and quantitative estimation of drug-likeness (**b**) for generated CB2 molecules.

Supplementary Figure S12. Using MACCS fingerprints-based t-SNE analysis to compare generated molecules, known CB2 ligands, and initial half-million training compounds

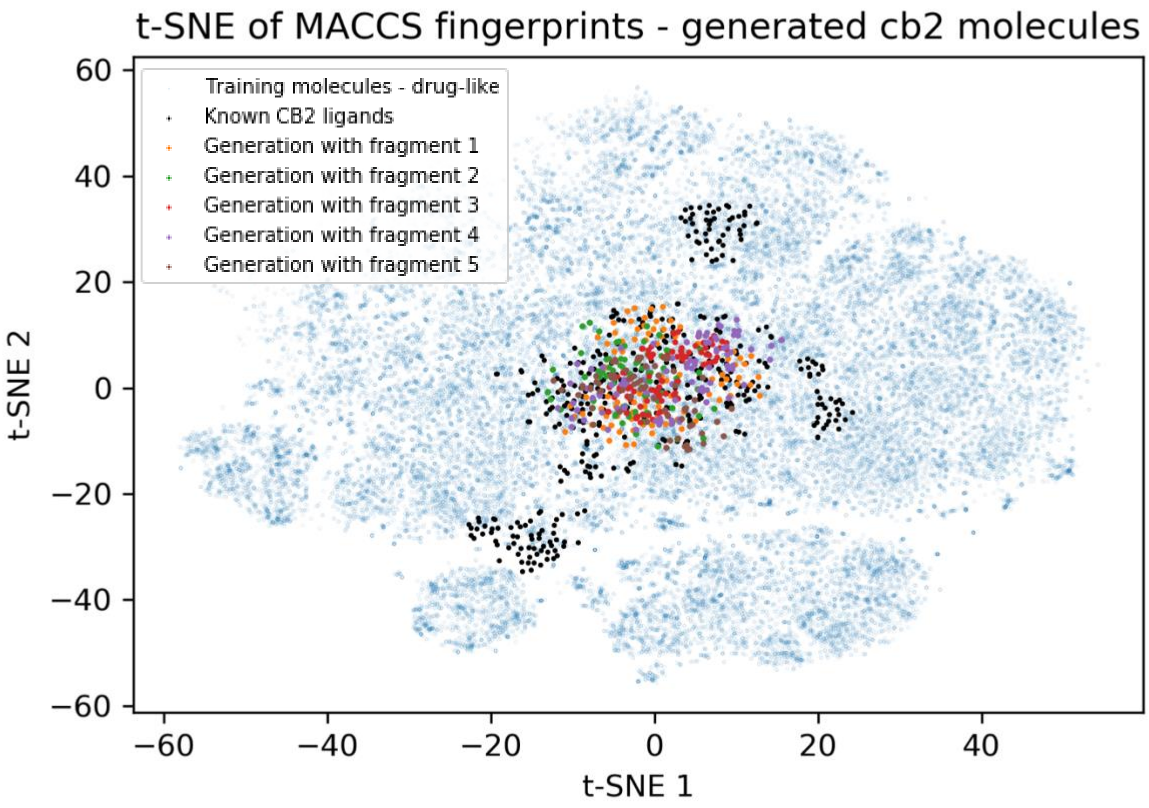

Supplementary Figure S13. Distribution and correlation of molecular weight (M.W.), topological polar surface area (TPSA), molecular refractivity (SMR), and Log of the octanol/water partition coefficient (SlogP) for known CB2 ligands and t-DeepMGM generated molecules

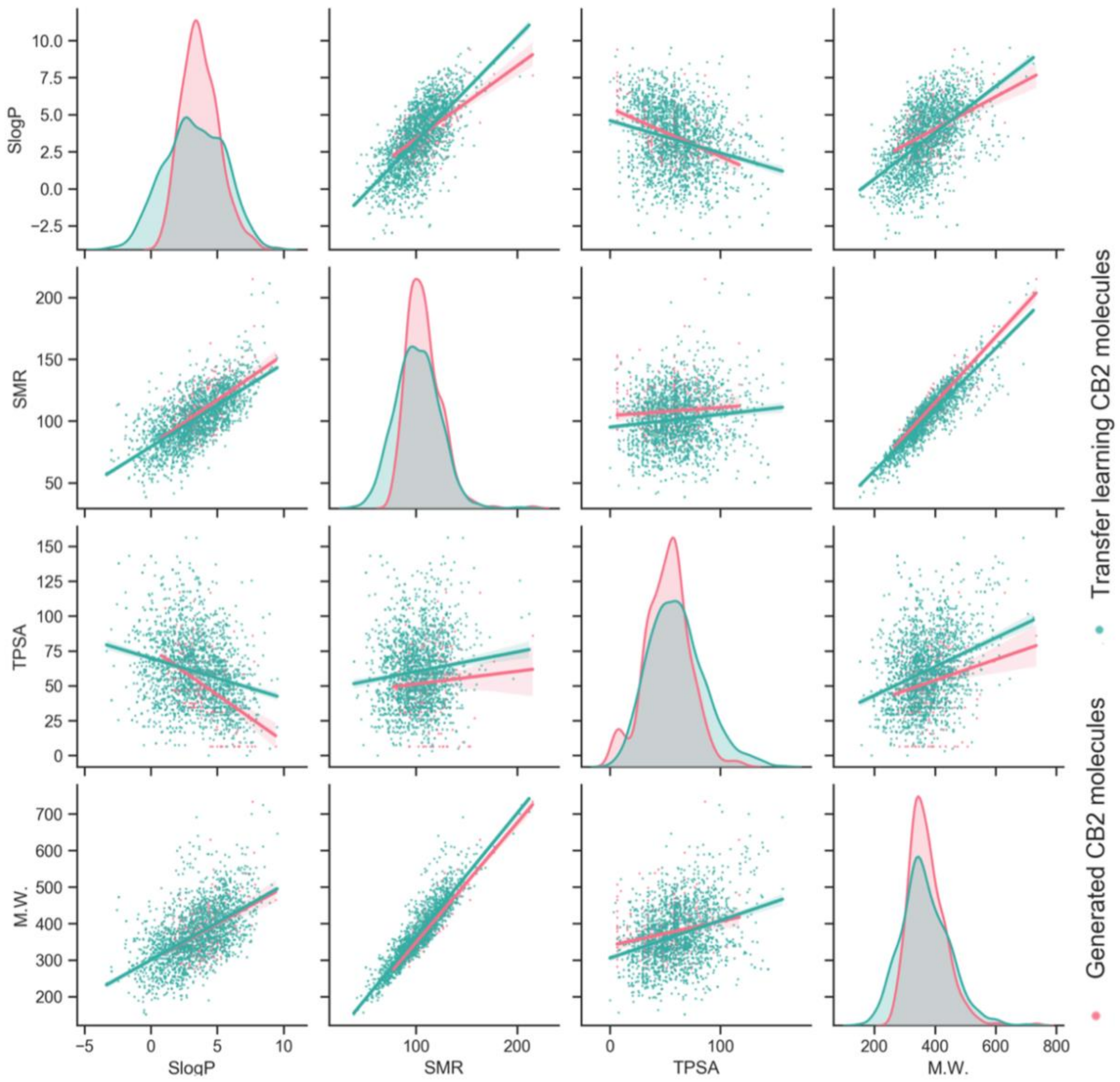

Supplementary Figure S14. ROC curves for the six-fold cross-validation of the established MLP Discriminator

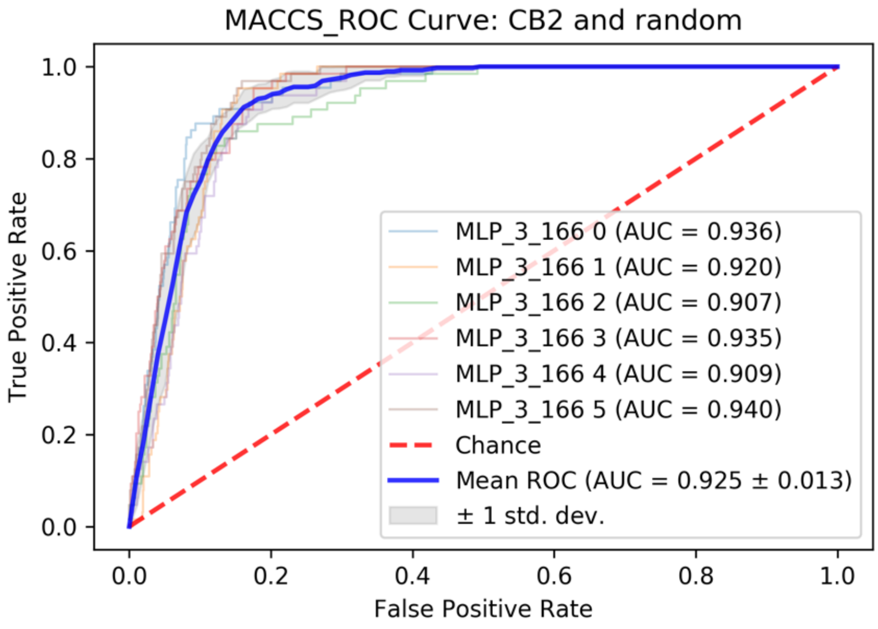

Supplementary Figure S15. The radar plot of the metrics for the MLP Discriminator

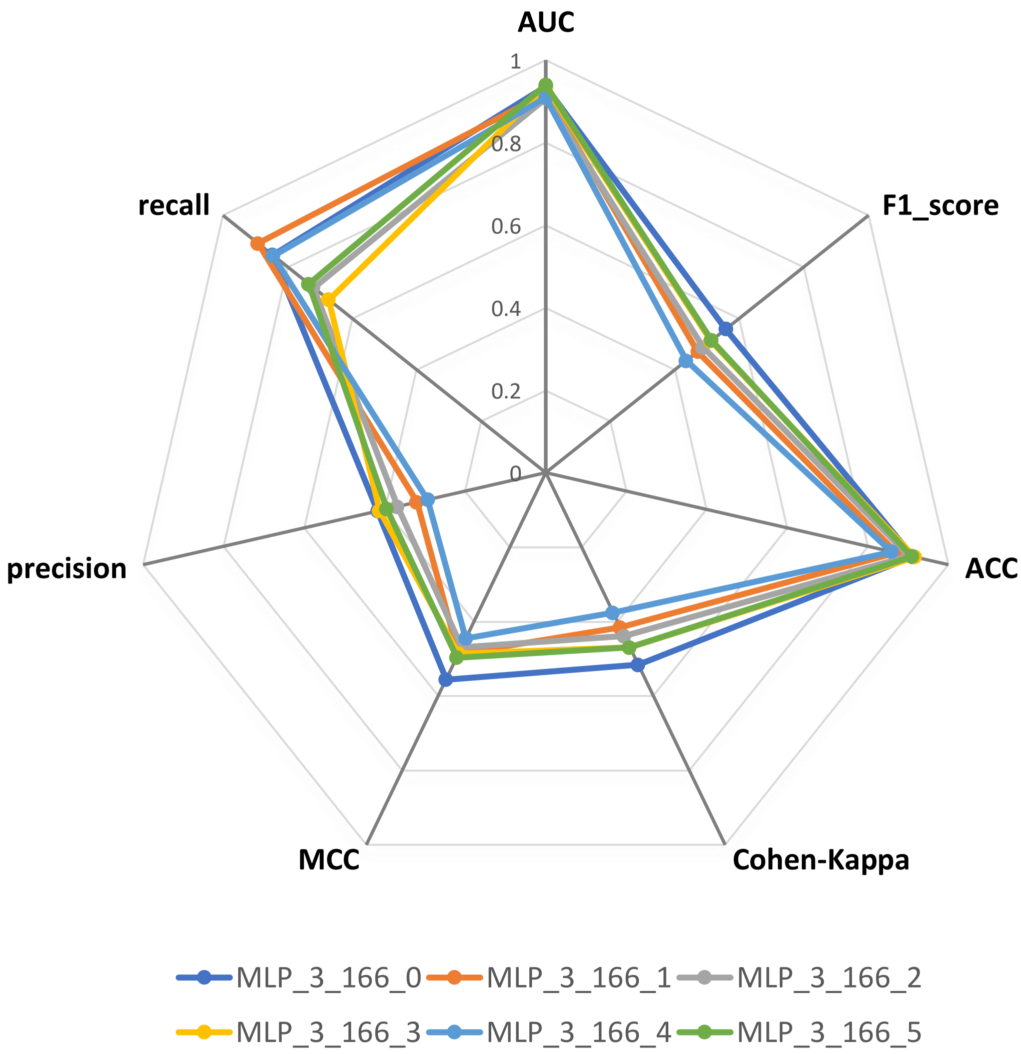

Calculated Metrics

Area under the ROC curve (AUC) was calculated with auc() after true positive rate and false positive rate were acquired with roc_curve(). AUC computes the area under the receiver operating characteristic (ROC) curve using the trapezoidal rule. AUC can be referred to indicate the performance of the model on separating classes.

Balanced F-score or F-measure (F1 score) was calculated with f1_score(). The F1 score can be interpreted as the weighted average of the precision and recall. The precision and recall have a relatively equal contribution to the F1 score. F1 = 2 * (precision * recall) / (precision + recall).

Accuracy classification score (ACC) was calculated with accuracy_score(). ACC computes subset accuracy as to whether the label predicted for one sample matches the corresponding true value.

Cohen's kappa was calculated with cohen_kappa_score(). Cohen's kappa measures inter-annotator agreement, which expresses the level of agreement between two annotators on a classification problem.

Matthew's correlation coefficient (MCC) was calculated with Matthews_corrcoef(). MCC is used to measure the quality of binary and multiclass classifications. It is a balanced measure that both the true and false positives and negatives are considered.

Precision was calculated with precision_score(). The precision measures the ability of a model not to label a negative sample as positive. Precision = true positives / (true positives + false positives).

Recall was calculated with recall_score(). The recall measures the ability of a model to find out all the positive samples. Recall = true positives / (true positive + false negative).

Supplementary Figure S16. ^{1}H NMR for XIE9-1-37

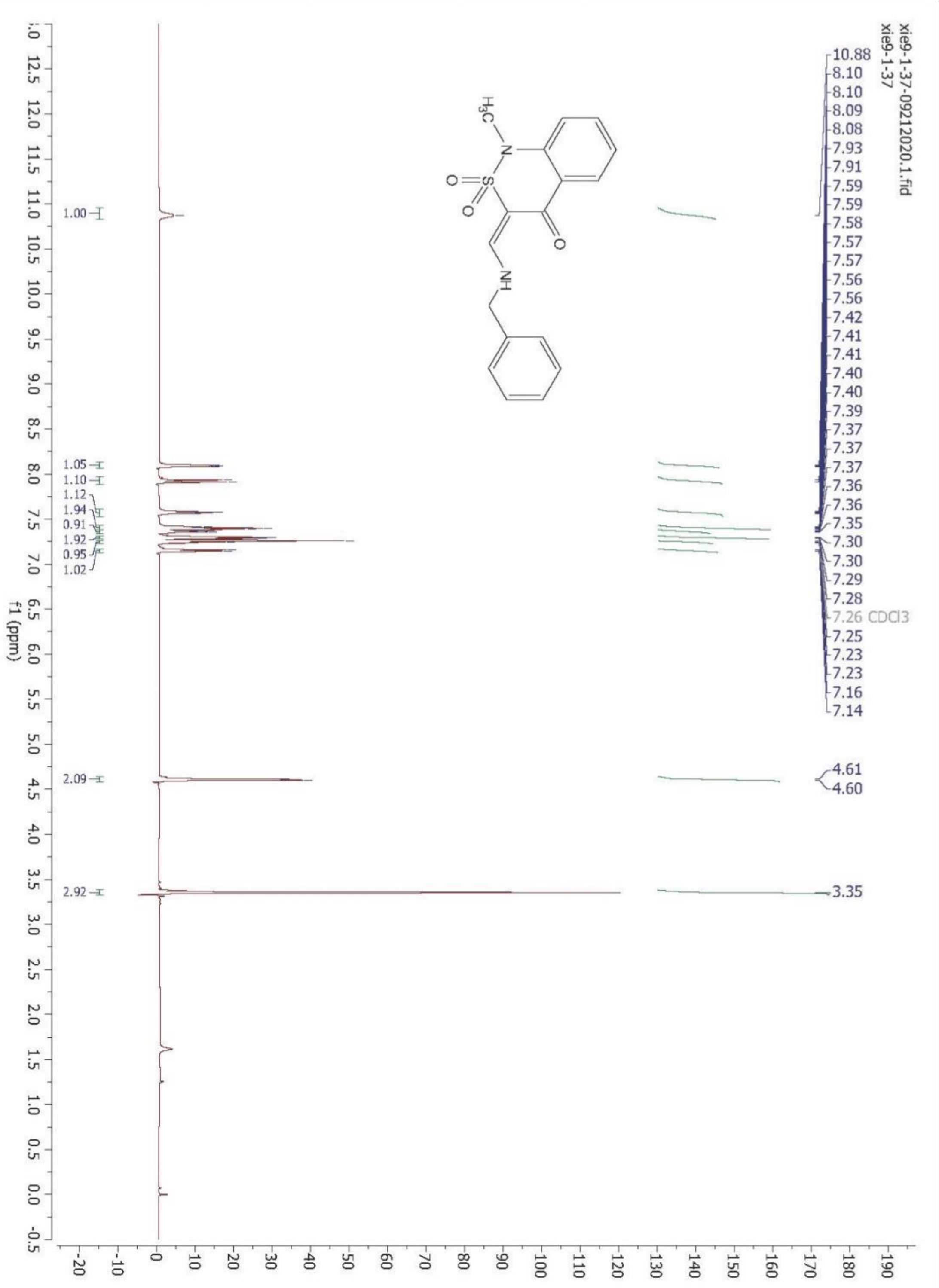

Supplementary Figure S17. ^{13}C NMR for XIE9-1-37

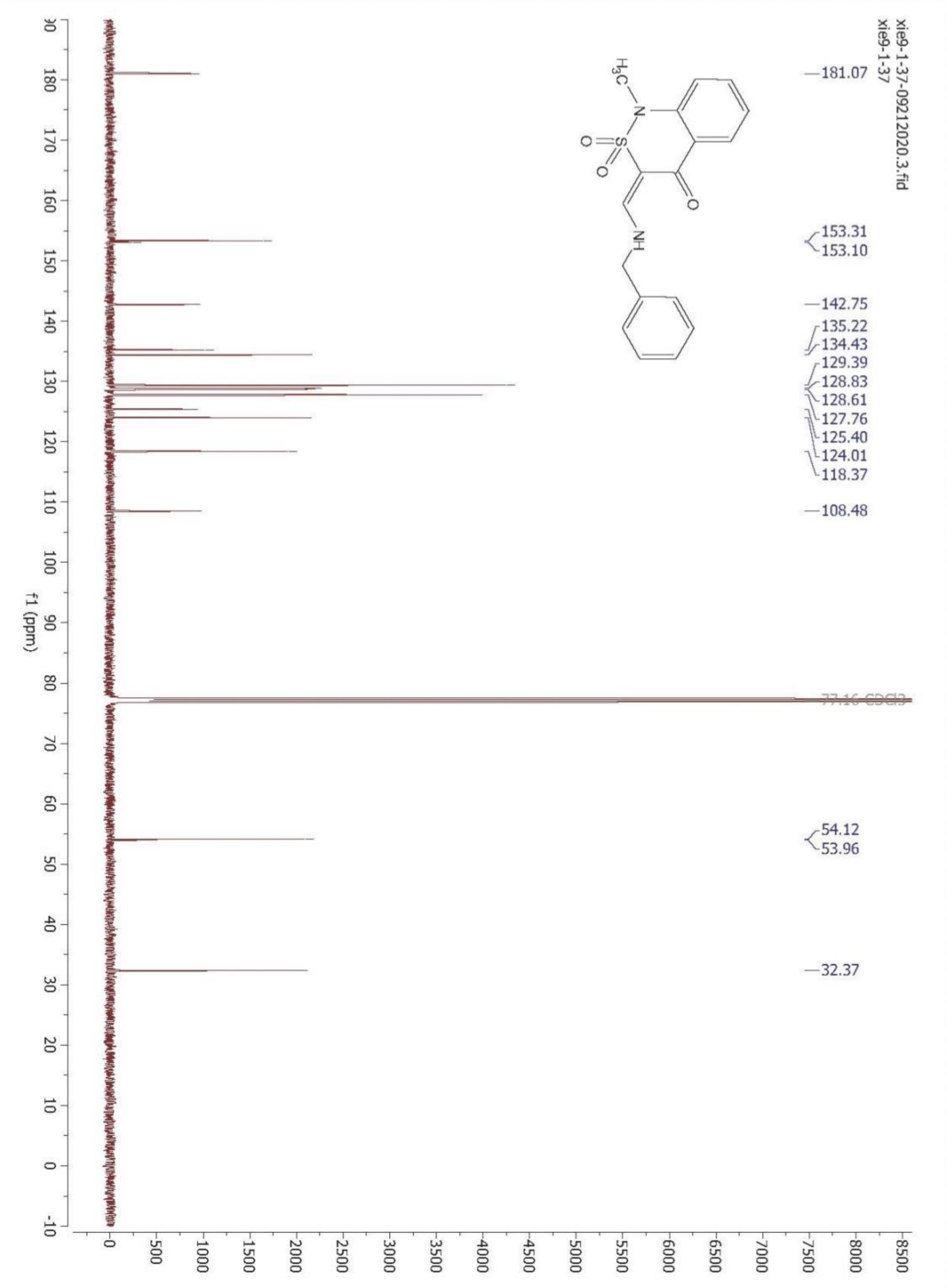

Supplement: Supplementary file 1 [file cells-11-00915-s001.zip › cells-1593707-supplementary.pdf]
